# A Cytoplasmic Heme Sensor Illuminates the Impacts of Mitochondrial and Vacuolar Functions and Oxidative Stress on Heme-Iron Homeostasis in Cryptococcus neoformans

**DOI:** 10.1128/mBio.00986-20

**Published:** 2020-07-28

**Authors:** Gaurav Bairwa, Eddy Sánchez-León, Eunsoo Do, Won Hee Jung, James W. Kronstad

**Affiliations:** a Michael Smith Laboratories, Department of Microbiology and Immunology, University of British Columbia, Vancouver, British Columbia, Canada; b Department of Systems Biotechnology, Chung-Ang University, Anseong, Republic of Korea; Yonsei University

**Keywords:** macrophage, heme sensor, mitochondria, vacuole, artemisinin, metformin, reactive oxygen species, biosensors, heme-related drugs

## Abstract

Pathogens must compete with hosts to acquire sufficient iron for proliferation during pathogenesis. The pathogenic fungus Cryptococcus neoformans is capable of acquiring iron from heme, the most abundant source in vertebrate hosts, although the mechanisms of heme sensing and acquisition are not entirely understood. In this study, we adopted a chromosomally encoded heme sensor developed for Saccharomyces cerevisiae to examine cytosolic heme levels in C. neoformans using fluorescence microscopy, fluorimetry, and flow cytometry. We validated the responsiveness of the sensor upon treatment with exogenous hemin, during proliferation in macrophages, and in strains defective for endocytosis. We then used the sensor to show that vacuolar and mitochondrial dysregulation and oxidative stress reduced the labile heme pool in the cytosol. Importantly, the sensor provided a tool to further demonstrate that the drugs artemisinin and metformin have heme-related activities and the potential to be repurposed for antifungal therapy. Overall, this study provides insights into heme sensing by C. neoformans and establishes a powerful tool to further investigate mechanisms of heme-iron acquisition in the context of fungal pathogenesis.

## Abstract (precis)

Invasive fungal diseases are increasing in frequency, and new drug targets and antifungal drugs are needed to bolster therapy. The mechanisms by which pathogens obtain critical nutrients such as iron from heme during host colonization represent a promising target for therapy. In this study, we employed a fluorescent heme sensor to investigate heme homeostasis in Cryptococcus neoformans. We demonstrated that endocytosis is a key aspect of heme acquisition and that vacuolar and mitochondrial functions are important in regulating the pool of available heme in cells. Stress generated by oxidative conditions impacts the heme pool, as do the drugs artemisinin and metformin; these drugs have heme-related activities and are in clinical use for malaria and diabetes, respectively. Overall, our study provides insights into mechanisms of fungal heme acquisition and demonstrates the utility of the heme sensor for drug characterization in support of new therapies for fungal diseases.

## INTRODUCTION

Invasive diseases caused by fungal pathogens are increasing in frequency, and current antifungal therapies are inadequate (1–5). One of these fungal pathogens, the encapsulated yeast Cryptococcus neoformans, causes pulmonary cryptococcosis and cryptococcal meningoencephalitis in immunocompromised individuals and organ transplant patients (6, 7). Nearly 300,000 cases of cryptococcal meningoencephalitis are reported yearly leading to ∼200,000 deaths, with the highest occurrence in Sub-Saharan Africa (8, 9). Disturbingly, cryptococcal disease accounts for ∼15% of deaths in HIV-AIDS patients despite advances in antiretroviral therapy, and there is a desperate need for new antifungal therapies. Another species, Cryptococcus gattii, has emerged as a deadly pathogen for immunocompetent individuals in the Pacific Northwest region of the United States and Vancouver Island in Canada (10–12). C. neoformans and C. gattii share major virulence traits, including elaboration of a capsule and melanin, secretion of extracellular enzymes, and the ability to grow at 37°C (6, 13–15).

Iron acquisition is critical for fungal and other microbial pathogens to proliferate and cause disease (16–18). Vertebrate hosts restrict access to iron to prevent the growth of microbial pathogens in a process termed nutritional immunity, and this response is a critical factor in determining the outcome of disease progression for bacterial and fungal pathogens (16, 19–21). Most iron in vertebrate hosts is present in heme, and heme-containing proteins such as hemoglobin and the haptoglobin-hemoglobin complex are potentially abundant sources of iron for fungal pathogens during disease (18). The mechanisms for acquisition and use of heme-iron sources are starting to be discovered in some fungi, including Aspergillus fumigatus, Candida albicans, Candida glabrata, C. neoformans, Schizosaccharomyces pombe, and Paracoccidioides brasiliensis (22–33). For example, studies in C. neoformans revealed important roles in heme use for endocytosis and endomembrane trafficking machinery, including the ESCRT proteins Vps22, Vps23, and Snf7, and proteins for clathrin-mediated endocytosis such as Chc1 (clathrin heavy chain 1), Las17 (a nucleation‐promoting factor for actin assembly), and Rvs161/Rvs167 (vesicle scission proteins; amphiphysins) (25, 26, 28). A recent study also highlighted a role for the SNARE regulator Vps45 in iron use from heme (34). Furthermore, a hemophore encoded by *CIG1* is unique to C. neoformans and is involved in heme use and virulence (35). Other proteins that are involved in direct binding of heme or hemoglobin in other fungi, such as the CFEM hemophore proteins Rbt5 and Pga7 in C. albicans (30), have not been identified in C. neoformans. Interestingly, the CFEM hemophore proteins in C. albicans constitute a relay network that has recently been shown to mediate heme acquisition from human serum albumin (36). For C. neoformans, defects in cellular trafficking for heme uptake result in attenuated virulence, highlighting the importance of heme iron in pathogenesis (25, 26, 35). Many of the iron acquisition functions in C. neoformans are regulated by iron regulatory proteins such as the monothiol glutaredoxin Grx4 and the transcription factors Cir1 and HapX (37–39).

**FIG 1:**
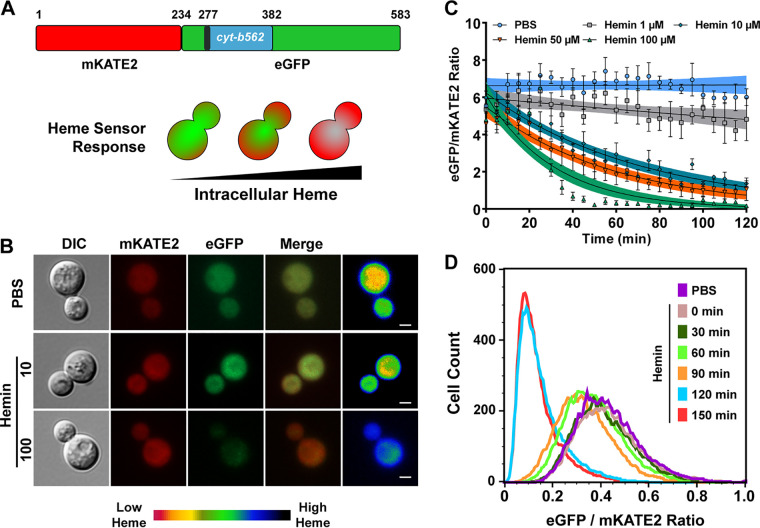
Characterization of C. neoformans cells expressing a heme sensor (CnHS). (A) Schematic diagram of the mKATE2-cytochrome *b*_562_-eGFP fluorescent heme sensor protein (CnHS) and depiction of the response (green to red) of the CnHS in a yeast cell with increasing intracellular heme levels. (B) Wide-field fluorescence microscopy of WT*^hs^* cells expressing the cytosolic CnHS. Iron-starved cells were incubated with hemin (10 and 100 μM) for 45 min at 30°C, and eGFP and mKATE2 fluorescence was observed and captured. Images are representative of a minimum of three independent experiments. The heat map shows the ratio of mean intensity values of eGFP and mKATE2 fluorescence generated in ImageJ 1.52q and represented as a pseudocolored image (far right, 14-color red-green blue [RGB] look-up table [LUT]); red and black are equivalent to low and high intracellular heme levels, respectively. Bars, 2 μm. (C) Dynamic changes in eGFP/mKATE2 fluorescence ratios of the CnHS in WT*^hs^* cells incubated with the indicated concentrations of hemin. Fluorescence from eGFP and mKATE2 was measured in a black 96-well plate using a Tecan Infinite 200 microplate reader, and the data were plotted as the ratios of eGFP and mKATE2 fluorescence after normalization with the background fluorescence of WT cells without the heme sensor. The results are the averages from three to six independent experiments ± standard errors of the means (SEMs), with the solid lines representing the nonlinear exponential regression analysis for the data points at each hemin concentration. (D) Flow cytometry analysis demonstrating the changes in eGFP/mKATE2 ratios of the WT*^hs^* cells incubated with hemin (100 μM) for the indicated times. The analysis was performed with a population of mKATE2-positive gated cells and is representative of three independent experiments.

Despite these advances, mechanistic insights into the processes for heme acquisition, intracellular trafficking, and storage are rudimentary for C. neoformans and other fungal pathogens. One of the major limitations in understanding these processes is the lack of molecular heme sensors that can detect the presence of heme in real time in response to factors that perturb heme acquisition and trafficking. It is also important to distinguish the fraction of heme associated with proteins from the fraction that is freely available as the labile heme pool in various cell compartments. A key recent advance is the use of cytosolic or organelle-specific fluorescent resonance energy transfer (FRET)-based molecular fluorescent heme sensors to detect intracellular labile heme (40–42). For example, a genetically encoded, fluorescent heme sensor (HS1) was recently developed that consists of the far-red fluorescent protein mKATE2 fused to a split green fluorescent protein (eGFP) with an embedded heme-binding domain from bacterial cytochrome *b*_562_ (Cyt *b*_562_) (Fig. 1A) (41). Heme binding to the Cyt *b*_562_ domain quenches the eGFP fluorescent signal (42). This sensor provides a robust tool to study heme trafficking and internal heme physiology in eukaryotic cells as demonstrated in Saccharomyces cerevisiae and mammalian cells (41, 43).

In the present study, we constructed C. neoformans strains expressing a codon-optimized version of the heme sensor developed for S. cerevisiae (41). We employed the strains to investigate the impact on heme sensing of mutations in genes for uptake functions and clathrin-mediated endocytosis and in response to an inhibitor of endocytosis, chlorpromazine. Experiments with the sensor strains also revealed that phagocytosis by a macrophage-like cell line results in reduced cytosolic heme levels in internalized yeast cells. Mitochondrial and vacuole functions and oxidative stress were also found to influence the cytosolic heme pool. Furthermore, we used the strains with the heme sensor to demonstrate that drugs (artemisinin and metformin) proposed to have heme-related activities have negative impacts on the cytosolic heme pool and fungal proliferation. Overall, our analyses provide insights into heme homeostasis for a fungal pathogen in response to conditions relevant to the host environment and antifungal therapy.

## RESULTS

### Characterization of a cytosolic heme sensor for C. neoformans.

To analyze heme uptake and factors influencing the cytosolic heme pool in C. neoformans, we constructed a strain expressing a codon-optimized version of the genetically encoded mKATE2-Cyt *b*_562_-eGFP fluorescent sensor designed for S. cerevisiae (41) (Fig. 1A). We used the variant HS1-M7A in which heme binding affinity is responsive to the cytosolic labile heme pool (41), and we expressed the heme sensor gene under the control of the elongation factor promoter 1 (*p*EF1). Initially, the utility of the heme sensor (designated CnHS) was validated by performing a qualitative microscopy analysis of eGFP and mKATE2 fluorescence in wild-type (WT) strains expressing the sensor (designated WT*^hs^*). As expected, we observed basal eGFP and mKATE2 fluorescence in the cytosol of WT*^hs^* cells incubated in phosphate-buffered saline PBS as a control (Fig. 1B). Incubation of WT*^hs^* cells with hemin (ferric chloride heme) for 45 min resulted in a significant quenching of the eGFP fluorescence, indicating internalization of hemin (Fig. 1B). Importantly, quenching of the eGFP fluorescence increased with hemin concentration (i.e., 10 μM versus 100 μM), and no significant change in the fluorescence of mKATE2 was observed at either hemin concentration (Fig. 1B). We further confirmed the responsiveness to hemin by using fluorescence microscopy to quantitate the eGFP/mKATE2 ratios in >50 cells per condition (see Fig. S1 in the supplemental material). Overall, the observed reduction in the ratio of eGFP to mKATE2 fluorescence indicated that the heme sensor is responsive to changes in cytosolic heme levels upon incubation with hemin.

10.1128/mBio.00986-20.1 FIG S1 The CnHS heme sensor in C. neoformans responds to extracellular hemin in time- and concentration-dependent manners. Heme-dependent change in eGFP/mKATE2 fluorescence ratios of the CnHS in iron-starved WT*^hs^* cells incubated with and without hemin (10 and 100 μM) at 30°C for 0 and 45 min. Fluorescence values were determined using wide-field fluorescence microscopy observations of the cells (*n* > 50). The data are representative of at least three independent experiments. ****, *P* < 0.0001, one-way ANOVA followed by Tukey’s HSD *post hoc* test. Download FIG S1, PDF file, 0.1 MB. Copyright © 2020 Bairwa et al. 2020 Bairwa et al. This content is distributed under the terms of the Creative Commons Attribution 4.0 International license.

We next examined the dynamics of hemin uptake in C. neoformans by performing fluorimetric analyses to monitor time-dependent responses of the heme sensor. Iron-starved cells were incubated with different concentrations of hemin (0 to 100 μM), and changes in the ratio of eGFP to mKATE2 fluorescence were monitored at regular intervals. As shown in Fig. 1C and S2, the eGFP/mKATE2 fluorescence ratio remained stable in the PBS control (no hemin) for the duration of the fluorimetric observations. In contrast, a gradual reduction in the eGFP/mKATE2 ratio was observed over time due to a decrease in the eGFP signal in response to different concentrations of hemin (Fig. 1C). The decline in the eGFP/mKATE2 ratio was faster at higher concentrations of hemin (e.g., 50 and 100 μM), although we did observe a lag in the responsiveness. Importantly, the highest concentration of hemin (100 μM) completely quenched eGFP fluorescence by 1 h (Fig. 1C). Notably, no significant changes in the level of fluorescence of mKATE2 were observed under any of the conditions (Fig. S2). The response of the heme sensor was not altered upon incubation of cells at different temperatures (30°C versus 37°C) or across a pH range (4.0 to 8.0) (G. Bairwa and E. Sánchez-León, unpublished data). Changes in the basal fluorescence of either eGFP or mKATE2 were also not observed in the presence of FeCl_3_ as the sole inorganic iron source, validating the specificity of the sensor for heme (see Fig. S3A). In contrast, coincubation of cells with both FeCl_3_ and hemin resulted in a slower heme sensor response with increasing concentration of FeCl_3_, suggesting that the presence of inorganic iron slowed heme uptake or interfered with sensing in WT*^hs^* cells (Fig. S3B). To examine the influence of iron starvation and nutrient availability in more detail, we measured eGFP and mKATE2 fluorescence in cells grown in rich and low-iron media. The cells grown in low-iron medium had a higher eGFP/mKATE2 ratio than the cells grown in rich medium, suggesting that starvation resulted in a reduction of the intracellular heme pool (see Fig. S4A). To confirm this observation, we used a biochemical approach to measure the total heme content of the cells grown under either rich or low-iron medium conditions and found that the heme content was ∼2-fold less in the iron-starved cells (Fig. S4B).

10.1128/mBio.00986-20.2 FIG S2 Stability and responses of the fluorescence signals of eGFP and mKATE2 in WT*^hs^* cells. The fluorescence signals of eGFP and mKATE2 in WT*^hs^* cells incubated in PBS with and without hemin (100 μM) were monitored in a black 96-well plate using a Tecan Infinite 200 microplate reader for the indicated time points. The data were plotted as relative fluorescence units (RFU) of eGFP and mKATE2 fluorescence after normalization with the background fluorescence of WT cells without the heme sensor. The results are the averages from three independent experiments, with the standard errors of the means shown by the bars (not visible for mKATE2 because of the small variation). Download FIG S2, PDF file, 0.2 MB. Copyright © 2020 Bairwa et al. 2020 Bairwa et al. This content is distributed under the terms of the Creative Commons Attribution 4.0 International license.

10.1128/mBio.00986-20.3 FIG S3 Inorganic FeCl_3_ effect on heme sensor response. (A) eGFP/mKATE2 fluorescence ratios of the CnHS in WT*^hs^* cells incubated with the indicated concentrations of FeCl_3_ (0 to 100 μM). The fluorescence levels for eGFP and mKATE2 were recorded every 5 min and are shown at the 30-min time point. The data were analyzed by averaging the ratios of all the eGFP and mKATE2 fluorescence values and represent the averages from two independent experiments ± SDs. (B) eGFP/mKATE2 fluorescence ratios of the CnHS in WT*^hs^* cells upon incubation for 2 h with different combinations of FeCl_3_ (0 to 100 μM) and hemin (0 to 100 μM). The heat map scale depicts the color representation of the eGFP/mKATE2 values for the range of 4.5 to 9.5. Download FIG S3, PDF file, 0.1 MB. Copyright © 2020 Bairwa et al. 2020 Bairwa et al. This content is distributed under the terms of the Creative Commons Attribution 4.0 International license.

10.1128/mBio.00986-20.4 FIG S4 CnHS response and total intracellular heme levels in rich and low-iron media. (A) Response of CnHS in WT*^hs^* cells incubated in rich (YPD) and defined low-iron medium (dLIM) with BPS at 30°C for 16 and 3 h, respectively. The data correspond to changes of eGFP/mKATE2 ratios determined using fluorescence microscopy of the cells (*n* > 80) and are representative of three independent experiments with error bars showing ± SDs. ****, *P* < 0.0001, two-tailed Student’s *t* test. (B) Quantification of the total intracellular heme levels in C. neoformans wild-type strain grown in YPD and dLIM BPS as in panel A. The data represent the averages from three independent experiments ± SEMs. ****, *P* < 0.0001, two-tailed Student’s *t* test. Download FIG S4, PDF file, 0.1 MB. Copyright © 2020 Bairwa et al. 2020 Bairwa et al. This content is distributed under the terms of the Creative Commons Attribution 4.0 International license.

To further validate the responsiveness of the heme sensor, we performed flow cytometry on the WT*^hs^* cells incubated with hemin (100 μM) for different times. Approximately 30,000 mKATE2-positive cell events were analyzed for both eGFP and mKATE2 fluorescence signals, and we found that the bulk of the cell population shifted toward reduced eGFP/mKATE2 ratio in a time-dependent manner (Fig. 1D). Notably, the cell populations at earlier time points (up to 90 min) had a wider range of eGFP/mKATE2 ratios (0.2 to 0.8, median of 0.3 to 0.4) than cells at 120 and 150 min, when a large proportion of the cells had similar and lower eGFP/mKATE2 ratios (0.0 to 0.4) observed by a narrow and higher peak with median eGFP/mKATE2 ratio of ∼0.1 (Fig. 1D). These results suggest variability in the level of heme binding among cells in the population (as also observed by Hanna et al. [41]) at early times and that the eGFP fluorescence of the heme sensor was quenched to the maximum level in the majority of the cells in the population by 120 min. Taken together, our observations validate the responsiveness of the heme sensor to external hemin and support subsequent use of the sensor to interrogate mechanisms of heme uptake and factors that influence the cytosolic heme pool for C. neoformans.

**FIG 2:**
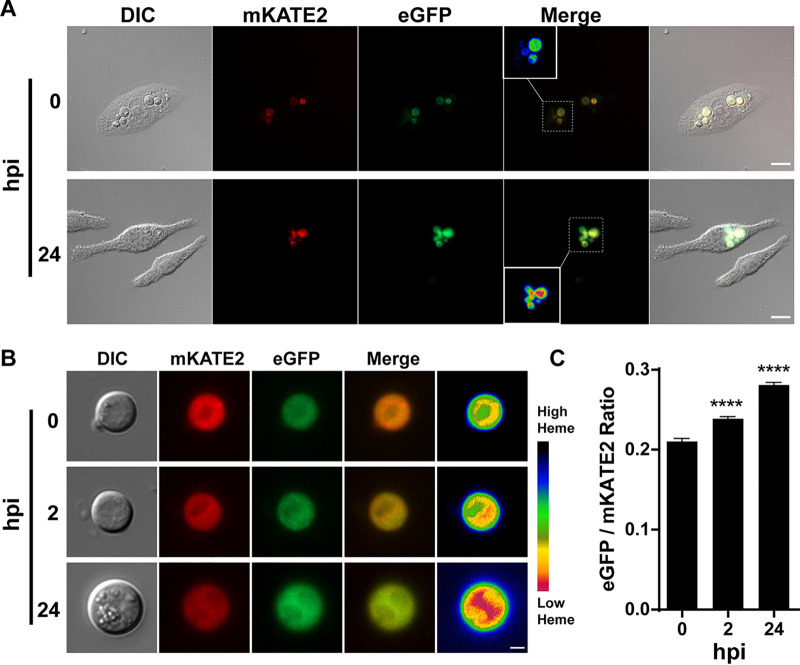
Phagocytosed C. neoformans cells show reduced cytosolic heme levels. (A) Wide-field fluorescence microscopy of WT*^hs^* cells internalized by J774A.1 murine macrophage-like cells. Images for DIC as well as eGFP and mKATE2 fluorescence were obtained at 0 and 24 h postinfection (hpi). Insets in the merge panels correspond to the heat map for the CnHS eGFP/mKATE2 fluorescence ratios as in Fig. 1B. Bars, 10 μm. Images are representative of two independent experiments (*n* > 70). (B) Wide-field fluorescence microscopy of WT*^hs^* cells (*n* > 80) isolated from J774A.1 murine macrophages at the indicated hours postinfection. Images are representative of a minimum of two independent experiments, with the heat map depicting heme levels as in Fig. 1B. Bar, 2 μm. (C) Quantitative analysis of the eGFP/mKATE2 fluorescence ratios of the CnHS from WT*^hs^* cells isolated from J774A.1 murine macrophages at the indicated hours postinfection. Fluorescence quantification was performed as for Fig. 1B with a minimum of 80 yeast cells. The results represent the averages from two to four independent experiments ± standard errors of the means (SEMs). ****, *P* < 0.0001, one-way analysis of variance (ANOVA) followed by Tukey’s honestly significant difference (HSD) *post hoc* test.

To further characterize the utility of the heme sensor, we used the WT*^hs^* strain to examine the influence of phagocytosis on the labile heme pool. Cells of the WT*^hs^* strain were phagocytosed by the murine alveolar macrophage-like cell line J774A.1, and eGFP and mKATE2 fluorescence was monitored in internalized yeast cells by comparing cells at 0 h with those at 24 h postinfection (Fig. 2A). We observed an increase in eGFP fluorescence, indicating a reduction of cytosolic heme levels in the internalized yeast cells. Due to potential autofluorescence from the macrophages, we also lysed the phagocytes and observed the internalized WT*^hs^* cells to further confirm and quantitate the change in the eGFP/mKATE2 ratio. The eGFP fluorescence from the resulting phagocytosed yeast cells showed a gradual increase between the initial cells at 0 h, the cells at 2 h, and the later 24-h stage of internalization (Fig. 2B and C). Importantly, within 2 h, the eGFP/mKATE2 ratio in WT*^hs^* cells increased by ∼15%, suggesting rapid establishment of a reduced heme condition for cells inside macrophages. By 24 h, the eGFP/mKATE2 ratio showed an increase of ∼35% compared to that at time zero (Fig. 2C). Overall, these data suggest that C. neoformans cells experience a reduction in the labile heme pool upon phagocytosis by macrophages and that the heme sensor strain has utility for interrogating interactions with host cells.

### Endocytic activity influences heme uptake and the cytosolic heme pool.

**FIG 3:**
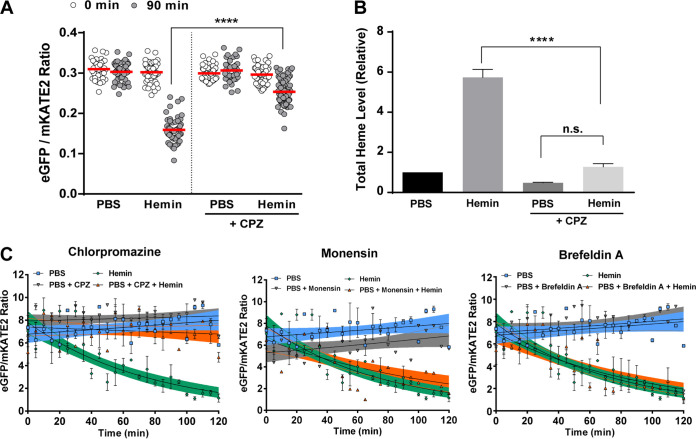
Endocytosis is required for heme uptake. (A) Changes in the eGFP/mKATE2 fluorescence ratios were determined using fluorescence microscopy of CnHS in WT*^hs^* cells incubated with hemin (100 μM) in the presence or absence of chlorpromazine (CPZ; 100 μM) at 30°C for 90 min. A total of 50 cells were analyzed, and the data are representative of three independent experiments. ****, *P* < 0.0001, one-way ANOVA followed by Tukey’s HSD *post hoc* test. (B) Quantification of total intracellular heme levels in the WT strain grown in YNB-Li medium with and without hemin (100 μM) in the presence and absence of CPZ (100 μM) for 1 h. The data represent the averages from three independent experiments ± SEMs. ****, *P* < 0.0001, one-way ANOVA followed by Tukey’s HSD *post hoc* test; n.s., not significantly different between the groups. (C) Dynamics of the changes in eGFP/mKATE2 fluorescence ratios versus time for the CnHS in WT*^hs^* cells incubated with hemin (100 μM) in the presence and absence of CPZ (100 μM) or the secretion inhibitor monensin (1.25 mg ml^−1^) or brefeldin A (50 μg ml^−1^) for the indicated times. Measurements were performed as for Fig. 1C, and the data represent the averages from three independent experiments ± SEMs.

Heme uptake in C. neoformans relies on clathrin-mediated endocytosis and on as-yet-unknown clathrin-independent mechanisms (26). To further examine the role of endocytosis in heme uptake and establish the utility of the heme sensor in studying heme internalization, we treated cells expressing the heme sensor (WT*^hs^*) with the endocytosis inhibitor chlorpromazine (CPZ). CPZ inhibits endocytosis by binding dynamin to impair the assembly of clathrin and is known to interfere with heme uptake in other organisms (44). CPZ treatment of WT*^hs^* cells resulted in a minimal reduction in the eGFP/mKATE2 ratio in response to hemin compared to that for the untreated condition (Fig. 3A). This result suggests that CPZ caused a defect in hemin uptake, and we confirmed this idea by measuring total heme content in iron-starved cells incubated for 1 h with hemin in the absence or presence of CPZ (Fig. 3B). We found that the total heme content of the cells incubated in the presence of hemin increased by ∼5-fold compared to that for the cells incubated without hemin, indicating uptake of external hemin. As expected, CPZ treatment prevented the uptake of hemin, resulting in no significant changes in the total cellular heme content (Fig. 3B). A time course of fluorimetry also revealed a reduced response of the heme sensor to exogenous hemin in the presence of CPZ (Fig. 3C). In contrast to CPZ, treatment of cells with inhibitors of intracellular trafficking, monensin and brefeldin A, did not cause any discernible effect on heme detection in the cytosol, as determined by the sensor response (Fig. 3C). Thus, inhibition of the secretory pathway did not influence the heme pool. We did note that monensin treatment alone reduced the eGFP/mKATE2 ratio at time zero relative to that of the PBS control, and this may reflect an influence of the inhibitor on fluorescence detection or the activity of the fluorescent proteins. Taken together, these results further support a role for clathrin-mediated endocytosis in hemin uptake by C. neoformans.

**FIG 4:**
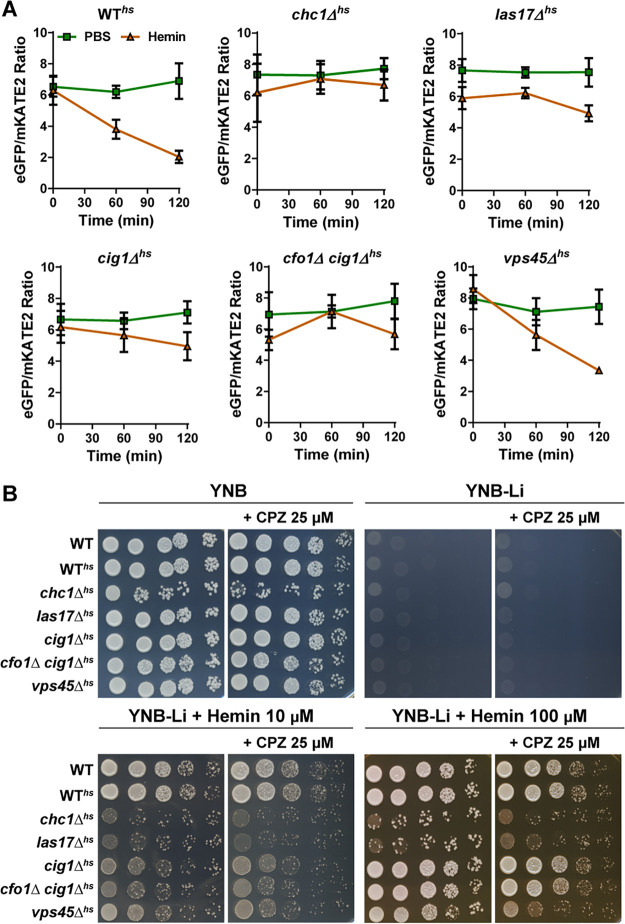
Defects in heme uptake and trafficking functions reduce the cytosolic heme pool. (A) Changes in eGFP/mKATE2 fluorescence ratios of the CnHS in the indicated strains grown in the presence or absence of hemin (100 μM) at 0, 60, and 120 min. The experiments were performed as for Fig. 1C. The data represent the averages from three independent experiments ± SEMs. Note that the differences in the ratios for the WT strain and the *vps45* mutant at time zero are not statistically significant. (B) Spot assays of 10‐fold serial dilutions of the indicated strains on medium supplemented as shown. Cells were starved for iron for 48 h prior to spotting. The plates were incubated for 4 days at 30°C before being photographed. YNB-Li is low-iron YNB medium supplemented with bathophenanthroline disulfonate (150 μM), CPZ; chlorpromazine.

Deletion of genes involved in clathrin-mediated endocytosis (i.e., *CHC1*, *LAS17*, *RVS161*, and *RVS167*) results in defective growth on hemin as an iron source for C. neoformans (26). To specifically assess hemin uptake, we expressed the CnHS sensor in the mutants lacking *CHC1* and *LAS17*, mutants lacking *CIG1* or *CIG1* and *CFO1* (*CIG1* encodes a hemophore and *CFO1* encodes a high-affinity ferric oxidoreductase), or a mutant defective in *VPS45* (encoding a regulator of vesicle trafficking and fusion [34]). In contrast to the WT strain, the mutants lacking Chc1 or Las17 showed no reduction in the eGFP/mKATE2 fluorescence ratio in response to hemin by 2 h of incubation, indicating a defect in hemin uptake (Fig. 4A). Furthermore, the mutants lacking Cig1, either alone or in combination with Cfo1, also showed less reduction in the eGFP/mKATE2 ratio than the WT*^hs^* strain, suggesting defective hemin uptake (Fig. 4A). The reduced eGFP/mKATE2 values at time zero in the mutants may indicate the presence of a compensatory heme uptake mechanism that is independent of clathrin-mediated endocytosis. We previously obtained evidence for more than one heme uptake mechanism in our studies of Cig1 and Chc1 (26, 35). Notably, the heme sensor response to the external hemin in the *vps45Δ^hs^* mutant was only slightly reduced in comparison to that of the WT*^hs^* strain, suggesting a modest influence on uptake. These data highlight the direct involvement of clathrin-mediated endocytosis and the hemophore Cig1 in hemin uptake and also suggest that the role of Vps45 may be more important for intracellular heme trafficking than for uptake (34).

We confirmed the influence of endocytosis on heme uptake by comparing the growth of the WT strain and the WT*^hs^* strain on medium with hemin and CPZ with the growth of mutants shown to be defective in uptake. As shown in Fig. 4B, CPZ reduced the growth of the WT strains and the mutants lacking Cig1 (and Cig1+Cfo1) or Vps45 on hemin. The *chc1Δ* and *las17Δ* mutants showed a more marked growth defect on hemin with or without CPZ, although the impact of the inhibitor was evident when a higher concentration (100 μM) of hemin was supplied. The differences may reflect the ability of cells to take up CPZ or the timing of its inhibitory activity on solid medium. The equivalent growth of the WT and WT*^hs^* also demonstrated that expression of the sensor did not interfere with proliferation (Fig. 4B), and this was true for the strains in liquid medium (G. Bairwa and E. Sánchez-León, unpublished data). Overall, these data emphasize the role of endocytosis in hemin internalization and highlight the utility of the heme sensor in characterizing mutants defective for heme uptake and intracellular trafficking.

### Inhibition of vacuolar and mitochondrial functions impairs detection of heme in the cytosol.

**FIG 5:**
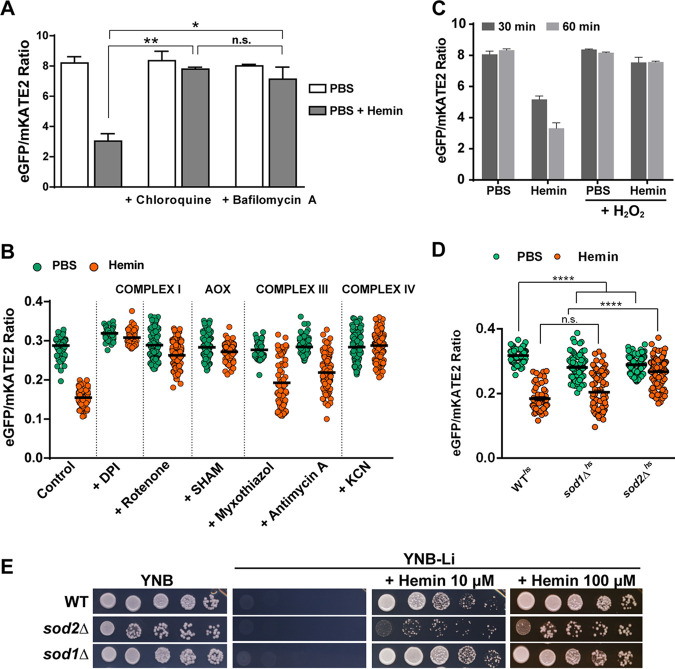
Mitochondrial and vacuolar functions and oxidative stress influence cytosolic heme levels. (A) Changes in eGFP/mKATE2 fluorescence ratios for the CnHS in WT*^hs^* cells incubated with and without hemin (100 μM) in presence or absence of the vacuole inhibitors chloroquine (100 μM) and bafilomycin A (1 μM). Measurements were performed as for Fig. 1C, and the data were analyzed by averaging the ratios of the eGFP and mKATE2 fluorescent values recorded every 5 min for 1 h. The data represent the averages from two independent experiments ± standard deviations (SDs). *, *P* < 0.05; **, *P* = 0.006, one-way ANOVA followed by Tukey’s HSD *post hoc* test; n.s., not significantly different between the groups. (B) Changes in eGFP/mKATE2 fluorescence ratios for the CnHS in WT*^hs^* cells incubated with and without hemin (100 μM) in the presence or absence of electron transport chain inhibitors: diphenyleneiodonium (DPI; 50 μM), rotenone (25 μM), salicylhydroxamic acid (SHAM; 10 mM), myxothiazol (5 μM), antimycin A (3 μg ml^−1^), or potassium cyanide (KCN; 10 mM). Measurements were determined using fluorescence microscopy of >50 cells followed by analysis as for Fig. 3A. Cells were incubated at 30°C (except at 37°C for DPI). The data are representative of 2 to 3 independent experiments. (C) The CnHS response in WT*^hs^* cells incubated with and without hemin (100 μM) in presence or absence of H_2_O_2_ (100 μM) for 30 or 60 min. The data were collected and analyzed as for panel A and represent the averages from three independent experiments ± SEMs. (D) The CnHS response in WT*^hs^*, *sod1Δ^hs^*, and *sod2Δ^hs^* strains incubated with and without hemin (100 μM) at 30°C for 45 min and determined by fluorescence microscopy (*n* > 100). The data are representative of three independent experiments. ****, *P* < 0.0001, one-way ANOVA followed by Tukey’s HSD *post hoc* test; n.s., not significantly different between the groups. Note that the sensor response for the two mutants is statistically different from WT in PBS. ****, *P* < 0.0001. (E) Spot assays of 10‐fold serial dilutions of the indicated strains on medium supplemented as shown.

We next employed the WT*^hs^* strain to investigate the importance of vacuolar and mitochondrial functions in regulating the labile heme pool in the cytosol. Several studies have demonstrated roles for the vacuole and mitochondria in the regulation of iron homeostasis (45–53). However, little is known about the roles of these organelles in the regulation of heme uptake and intracellular heme homeostasis in fungal pathogens. We therefore first analyzed the role of the vacuole by treating WT*^hs^* cells with chloroquine and bafilomycin A, inhibitors of organelle acidification. As shown in Fig. 5A (and described above), hemin causes a decrease in the eGFP/mKATE2 fluorescence ratio (∼2.5-fold) in WT*^hs^* cells. In contrast, incubation with either chloroquine or bafilomycin A impaired the response of the sensor to exogenous hemin, suggesting that vacuolar function (e.g., organelle acidification and/or an influence on trafficking) impacted the labile heme pool in the cytosol (Fig. 5A).

Mitochondria play a central role in heme metabolism by harboring part of the enzymatic machinery for heme biosynthesis as well as many heme-containing proteins (e.g., for the electron transport chain [ETC]). As an initial test of whether mitochondrial functions influenced the sensing of the labile heme pool, we examined the impact of ETC inhibitors on the response of the heme sensor. The contribution of complex I of the ETC was tested by exposing WT*^hs^* cells to diphenyleneiodonium (DPI) or rotenone, and eGFP/mKATE2 fluorescence was observed using fluorescence microscopy. Without treatment, WT*^hs^* cells showed a 50% reduction in the eGFP/mKATE2 ratio upon incubation with hemin (100 μM) for 45 min in comparison to the PBS control (Fig. 5B). In contrast, the inhibition of complex I led to a reduction in the labile heme pool in WT*^hs^* cells as observed by the eGFP/mKATE2 ratios, despite the presence of external hemin (Fig. 5B). We noted that DPI treatment both eliminated the response of the sensor to external hemin and elevated the basal fluorescence in the absence of hemin. The latter phenomenon was not observed with rotenone, and the difference may reflect additional inhibitory activities of DPI on flavoproteins and the generation of reactive oxygen species (ROS) (54). We next tested inhibition of alternative oxidase, a highly conserved inner mitochondrial membrane protein associated with virulence in C. neoformans and with stress responses in other fungal pathogens (55–59). Inhibition of the alternate oxidase by salicylhydroxamic acid (SHAM) in WT*^hs^* cells also negatively impacted the reduction of the eGFP/mKATE2 fluorescence ratio in the presence of external hemin, indicating an altered heme pool. We also asked whether inhibiting complex III activity influenced heme sensor activity (Fig. 5B). Antimycin A and myxothiazol, inhibitors of the cytochrome *c* reductase, prevent the transfer of electrons by complex III through inhibition of ubiquinol oxidation and the competitive inhibition of ubiquinol, respectively. Surprisingly, neither inhibitor had a significant impact on the eGFP/mKATE2 ratios of the WT*^hs^* cells upon incubation with hemin (100 μM) (Fig. 5B). In contrast to the observation for complex III, disruption of complex IV by inhibition of cytochrome *c* oxidase with potassium cyanide resulted in a marked defect in the response of the heme sensor. Overall, these results indicate that the integrity of the ETC and mitochondrial function have an impact on the labile heme pool in the cytosol.

### Oxidative stress reduces the cytosolic heme pool.

Given the observed impact of mitochondrial dysfunction, we hypothesized that oxidative stress and reactive oxygen species (ROS) resulting from impaired vacuolar and ETC function could influence the abundance and/or detection of the heme pool in the cytosol. In this context, several studies indicate a connection between mitochondrial dysfunction (e.g., by inhibiting ETC function), changes in the cellular redox state, and the generation of high levels of ROS (48, 53, 54, 60). To test this hypothesis, we measured the eGFP/mKATE2 ratio in WT*^hs^* cells grown with hemin in the presence or absence of hydrogen peroxide (H_2_O_2_), a potent inducer of oxidative stress. Coincubation of the WT*^hs^* cells with hemin and H_2_O_2_ abolished the response of the heme sensor such that no change in the eGFP/mKATE2 ratio was observed at 30 and 60 min (Fig. 5C). Importantly, and as shown earlier, the eGFP/mKATE2 ratio was reduced by 40% (30 min) and 60% (60 min) in the presence of hemin alone (Fig. 5C). We also investigated the impact of ROS by expressing the heme sensor in deletion mutants lacking either the cytosolic superoxide dismutase (strain *sod1*Δ*^hs^*) or the mitochondrial superoxide dismutase (strain *sod2*Δ*^hs^*). Superoxide dismutases, as well as other antioxidant proteins (e.g., catalases, glutathione peroxidases, and peroxiredoxins), protect cellular components from ROS damage (61, 62). We found that the *sod1*Δ^hs^ strain showed similar eGFP/mKATE2 fluorescence ratios to those of WT*^hs^* when exposed to hemin, and the *sod2*Δ*^hs^* strain exhibited a reduction in the sensor response under the same conditions (Fig. 5D). This result suggests that the antioxidant activity of mitochondrial Sod2 and ROS are important in regulating the cytosolic heme pool and the response of the sensor. As expected, the growth of the *sod2Δ* strain was significantly reduced on hemin-containing medium in comparison to that of the WT and *sod1*Δ strains, confirming a defect in the response to heme in the *sod2Δ* mutant. The defect could possibly be due to the higher mitochondrial oxidative stress or sensitivity to oxidative stress caused by heme (Fig. 5E). We did note that loss of either Sod1 or Sod2 resulted in lower eGFP/mKATE2 ratios in the PBS controls than in the WT, and this result may suggest that perturbation of the ability to respond to oxidative stress may increase the labile heme pool. Taken together, these results suggest an important contribution of oxidative stress in the regulation of heme homeostasis in C. neoformans.

### Drugs with heme-related activities cause dysregulation of the labile heme pool.

**FIG 6:**
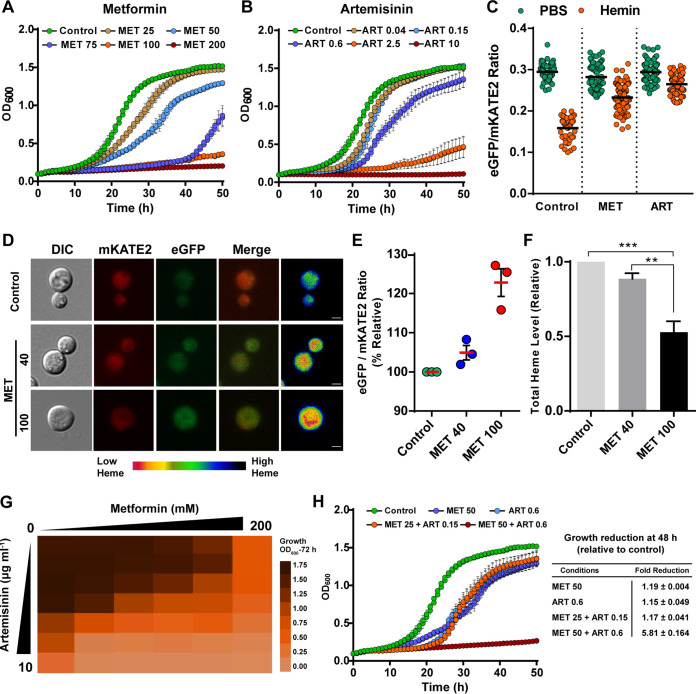
Metformin and artemisinin inhibit growth and influence the labile heme pool. Growth of the WT strain in YPD medium with the indicated concentrations of metformin (MET) (A) or artemisinin (ART) (B) at 30°C. Growth was monitored by recording the OD_600_ every 30 min over 48 h in a 96-well plate using a Tecan Infinite 200 Pro microplate reader. The data represent the means ± standard deviations (SDs) from two independent experiments. (C) Change in the eGFP/mKATE2 fluorescence ratios of the CnHS in WT*^hs^* cells incubated at 30°C with and without hemin (100 μM) in presence or absence of either 40 mM MET or 1 μg ml^−1^ ART determined using fluorescence microscopy (*n* > 50); cells were analyzed as for Fig. 3A. (D) The effect of MET on the heme pool was analyzed by incubating WT*^hs^* cells with MET (40 or 100 mM) in YPD for 16 h and observing fluorescence by microscopy. The images are representative of a minimum of three independent experiments, with the heat map depicting varied intracellular heme levels as in Fig. 1B. Bars, 2 μm. (E) eGFP/mKATE2 fluorescence ratios of the CnHS in WT*^hs^* cells incubated with MET (40 mM or 100 mM) for 16 h. The data represent the eGFP/mKATE2 ratios relative to the control growth condition (YPD only) of three independent experiments, with error bars showing the SEMs. (F) Quantification of the total intracellular heme content in the WT strain grown in YPD with MET (40 mM or 100 mM) for 16 h relative to that in YPD without MET. The data represent the averages from three independent experiments ± SEMs. **, *P* < 0.01; ***, *P* < 0.001, one-way ANOVA followed by Tukey’s HSD *post hoc* test. (H) Checkerboard growth assay of the WT strain in YPD medium supplemented with different combinations of MET (0 to 200 mM) and ART (0 to 10 μg ml^−1^). Cultures were incubated at 30°C for 72 h, and the OD_600_ was recorded using a Tecan Infinite 200 Pro microplate reader; the data are depicted as a heat map of the average values from two independent experiments. The heat map scale depicts the color representation of the OD_600_ values for the range 0.00 to 1.75. (H) Growth of the WT strain in YPD medium with the indicated concentrations of MET and ART alone or in combination at 30°C was monitored at OD_600_. The relative reduction in growth is indicated in the table on the right. The data represent the means ± SDs from two independent experiments.

The availability of a heme sensor in C. neoformans provides an opportunity to investigate the potential antifungal activity and mechanisms of action of drugs that have heme-related activities. For example, metformin (MET) (1,1-dimethylbiguanide hydrochloride) is an FDA-approved drug for the treatment of type 2 diabetes mellitus, and MET also has antifungal activity (63–65). MET suppresses the production of heme in yeast cells, and the suppression involves functional mitochondria (66, 67). The activity of the antimalarial drug artemisinin (ART) may involve an antimitochondrial influence as well as the generation of ROS in a heme-dependent manner (68–70). ART is also reported to have antifungal activity (71–73). Considering that C. neoformans has the ability to grow on medium with high hemin levels and is proficient in taking up extracellular hemin as an iron source, we determined whether MET and ART influence hemin internalization as well as the growth and survival of cells. We first monitored the effect of MET and ART on growth, and found reduced proliferation with increasing concentrations of the drugs (Fig. 6A and B). Notably, MET was fungistatic at the highest concentration (200 mM) tested, while ART was fungicidal at 10 μg ml^−1^ (see Fig. S5). To determine whether MET and ART influenced heme physiology, we monitored the heme sensor response of WT*^hs^* cells to external hemin in the presence and absence of the drugs individually. As shown in Fig. 6C, the eGFP/mKATE2 ratio was reduced to ∼50% from its basal level in the WT*^hs^* cells incubated with external hemin (100 μM) alone. However, the presence of subinhibitory concentrations of either MET (40 mM) or ART (1 μg ml^−1^) caused ∼20% and 10% reductions in the eGFP/mKATE2 ratio from the basal level with hemin, respectively, suggesting an impact on the heme pool or sensor activity (Fig. 6C). Thus, MET and ART both have a negative impact on the heme pool, and this may potentially explain their ability to inhibit proliferation. As a control, we also treated the WT*^hs^* cells with amphotericin B and did not observe an influence on the response of the heme sensor to hemin (G. Bairwa and E. Sánchez-León, unpublished results).

10.1128/mBio.00986-20.5 FIG S5 Measurements of fungicidal and fungistatic activity of metformin and artemisinin for C. neoformans. WT and WT*^hs^* cells were inoculated at 0.1 OD_600_ in YPD supplemented with and without metformin (MET; 100 and 200 mM) or artemisinin (ART; 1 and 10 μg ml^−1^) at 30°C and 200 rpm. Survival was monitored by plating to determine CFU at 0, 16, and 48 h. The data represent the averages from three independent experiments ± SEMs. Download FIG S5, PDF file, 0.1 MB. Copyright © 2020 Bairwa et al. 2020 Bairwa et al. This content is distributed under the terms of the Creative Commons Attribution 4.0 International license.

Given the reported influence of MET on heme production (66), we examined the effects of the drug on the heme sensor response and heme levels in more detail. By microscopy, we observed a reduction in the cytosolic heme pool when WT*^hs^* cells were grown in 40 or 100 mM MET for 16 h, with the ratio of eGFP/mKATE2 increased by nearly 25% at the highest concentration of metformin compared to that under the control condition (Fig. 6D and E). As expected for an impact on the labile heme pool, the total intracellular heme content of the cells grown in the presence of 40 mM or 100 mM MET was decreased by ∼10% and ∼60%, respectively, compared to that of untreated cells (Fig. 6F).

We next analyzed the antifungal activity of the combination of MET and ART by measuring the growth of the WT strain in a checkerboard assay. As shown in Fig. 6G and H, lower concentrations of MET (25 mM) or ART (0.15 μg ml^−1^) alone had only a slight effect on the growth of WT cells. However, the combination of MET (25 mM) and ART (0.15 μg ml^−1^) reduced growth in a manner similar to that with high concentrations of MET (50 mM) or ART (0.6 μg ml^−1^) alone (Fig. 6G and H). Furthermore, although the WT strain was able to grow at high concentrations of 50 mM for MET or 0.6 μg ml^−1^ of ART, growth was significantly reduced (∼6-fold) when both MET (50 mM) and ART (0.6 μg ml^−1^) were present (Fig. 6G and H). Thus, the combination of the two drugs lowered the dose of each required to achieve growth inhibition. We also examined whether the ART derivative dihydroartemisinin (DHA), which is more soluble than ART, might show synergy with MET. In this case, we determined the MICs and fractional inhibitory concentrations (FICs) for the drugs and again found an additive or indifferent effect (see Fig. S6). The MIC values for MET and DHA were 55 to 60 mM and 28 to 30 μg ml^−1^, respectively. Taken together, these results confirm that MET and ART (and DHA) have antifungal activity and indicate the potential for combination therapy.

10.1128/mBio.00986-20.6 FIG S6 The combination of metformin and the artemisinin derivative DHA exerts an additive or indifferent inhibitory effect on C. neoformans. Checkerboard MIC assay of the WT strain showing the fractional inhibitory concentration (FIC) indices of the combinatorial effect of MET (0 to 75 mM) and the ART derivative dihydroartemisinin (DHA; 0 to 30 μg ml^−1^). Cells were incubated in a 96-well plate with RPMI medium at ∼10^4^ cells ml^−1^ for 72 h at 37°C. The MIC values for MET and DHA were 55 to 60 mM and 28 to 30 μg ml^−1^, respectively. FIC indices were defined as follows: synergism, FIC of <0.5; additive, >0.5 to <1; indifferent, ≥1 to ≤2; antagonism, >4. The data represent the values from five independent experiments. Download FIG S6, PDF file, 0.1 MB. Copyright © 2020 Bairwa et al. 2020 Bairwa et al. This content is distributed under the terms of the Creative Commons Attribution 4.0 International license.

## DISCUSSION

Our understanding of heme sensing and the regulation of heme-iron acquisition by fungal pathogens is incomplete. Heme contains ∼80% of the iron in vertebrate hosts, and we previously demonstrated that heme is an important iron source for C. neoformans (35, 74). In the present study, we constructed a strain encoding a cytosolic heme sensor to address our goals of (i) understanding heme-iron acquisition during fungal proliferation and pathogenesis and (ii) identifying potential heme/iron-related targets for antifungal therapy. Initially, we validated the behavior of the heme sensor by demonstrating responsiveness to exogenous hemin by established microscopic, fluorimetric, and flow cytometry methods (41). Importantly, we demonstrated that the sensor detected the reduction in cytosolic heme levels expected to result from impaired endocytosis. Specifically, reduced cytosolic heme levels were found in cells treated with CPZ and in mutants such as the *cig1* and *las17* mutants that have lower heme uptake and attenuated virulence in mice (26, 35). The combination of pharmacological and genetic approaches supports a role for endocytosis in heme uptake, although we note that CPZ has also been reported to directly interact with heme (75). In general, the deployment of the heme sensor expands the opportunities to identify additional components for heme trafficking (e.g., receptors and heme-binding proteins) through mutant screens and to identify factors that impact heme homeostasis.

The heme sensor in C. neoformans was also responsive to intracellular conditions in phagocytes. Macrophages are key cells of the immune response against invading microbes such as C. neoformans (76). Macrophages also play central roles in removing excess heme resulting from hemolysis, in recycling iron from senescent erythrocytes, and in nutritional immunity (16, 77, 78). C. neoformans can avoid phagocytic killing and exploit macrophages to promote dissemination and movement across the blood brain barrier in a Trojan Horse strategy (79). Little is known about heme availability to fungal cells upon phagocytosis, and the use of the heme sensor demonstrated that cytosolic heme levels decreased between 2 h and 24 h of internalization. These results suggest that adaptation to the intracellular environment results in limited availability of exogenous heme and/or an adjustment of fungal heme homeostasis due to metabolic conditions in the phagolysosome. The strain harboring the heme sensor provides an opportunity for future work to examine the fungal response to manipulations that alter iron and heme availability in the phagolysosome (e.g., with macrophages lacking heme oxygenase, having impaired iron homeostasis, or upon drug treatment [80, 81]).

Our results also demonstrated that perturbing vacuolar and mitochondrial functions with inhibitors of acidification and the electron transport chain, respectively, impaired the responsiveness of the heme sensor to added hemin. In particular, vacuolar function may be centrally important in the use of heme iron by fungi, given earlier work demonstrating the importance of endocytosis for heme uptake in C. albicans (23). Studies in S. cerevisiae have also highlighted the roles of the vacuole and mitochondria in balancing iron homeostasis by regulating iron uptake and storage processes via transcriptional control of the genes encoding these functions (48, 49, 52, 82). In S. cerevisiae, defects in vacuolar acidification due to loss of V-ATPase activity result in fragmentation of mitochondria and loss of the energetic potential across the inner membrane. Mutants defective in the V-ATPase also have dysregulated intracellular pH and are sensitive to oxidizing agents (53, 82). Iron supplementation restores the mitochondrial phenotypes, and iron starvation appears to account for the mitochondrial defects. Additionally, part of the influence of vacuolar impairment on mitochondria in S. cerevisiae comes from defective compartmentalization of amino acids resulting in increased cytoplasmic amino acid pools that limit iron availability and cause mitochondrial defects (50). In particular, cysteine promotes iron limitation and causes oxidative stress in the context of impaired vacuolar ATPase function. It will be interesting in the future to investigate whether amino acids influence the response of the heme sensor in C. neoformans.

In the context of oxidative stress, we found that treatment of C. neoformans with hydrogen peroxide interfered with intracellular heme sensing in response to exogenous hemin. This is consistent with a role for ROS and oxidative stress as a component of the influence of vacuolar and mitochondrial dysfunction (60). However, it is possible that hydrogen peroxide could interact with heme directly to impair the response of the sensor (75). Additionally, increased ROS due to vacuolar and mitochondrial dysfunction could stimulate the expression of heme-containing proteins (e.g., catalase and peroxidases) to potentially drive heme into a protein-bound form that is no longer detectable by the sensor (83). Further support for an impact of ROS and oxidative stress comes from our demonstration that loss of mitochondrial Sod2 resulted in reduced cytosolic heme and impaired growth on hemin as an iron source. Sod2 is required for virulence in both C. neoformans and C. gattii (84, 85). Changes in ROS generation also occur upon inhibition of the ETC, and this may account in part for the impact of the inhibitors on heme sensing. However, detailed additional experiments on the timing and concentrations of inhibitor treatment are needed to understand the impact of inhibition at each step in the ETC on ROS, heme sensing, and heme uptake.

The influence of the vacuole on sensor detection of heme in the cytosol could also be due to an indirect impact on uptake and subsequent intracellular partitioning of heme via the endomembrane system. That is, reduced vacuolar acidification may interfere with endocytosis, the endosomal sorting complex required for transport (ESCRT) pathway, and/or vesicle fusion, and we have previously shown that these functions participate in heme uptake and trafficking (25, 26, 28, 34). In addition to an influence on uptake, treatment with chloroquine or bafilomycin A could also block the release of stored heme or the cycling of endocytosed heme through the vacuole to eventually reach the cytosol. These ideas are reminiscent of the known role for the lysosome in iron acquisition via endocytosis of the transferrin receptor and the role of the vacuolar heme exporter, Abc3, that delivers stored heme to the cytosol in the yeast S. pombe (86).

Our investigation of iron and heme acquisition in fungal pathogens is motivated by the need to identify new opportunities for antifungal therapy. We therefore employed the heme sensor in C. neoformans to obtain additional evidence that the FDA-approved drugs MET and ART have heme-related activities and could potentially be repurposed as antifungal agents. The mode of action of MET is somewhat controversial, but the drug is known to suppress hepatic glucose production and improve hyperglycemia in diabetics (66, 87, 88). The effects of the drug include positive influences on mitochondrial respiration and activation of the 5′ AMP-activated protein kinase. Recent work also indicates that MET suppresses heme production in yeast, erythrocytes, erythropoietic cells, and hepatocytes and that the drug acts to influence the redox states of heme (66). The same study and earlier work (88) indicated that ART had an even greater activity in suppressing heme levels. ART is a frontline drug for treating malaria, and it is generally used in combination therapy (72). Although a full understanding of the mode of action of ART is not known, strong evidence indicates that cleavage of the endoperoxide ring is required to convert the drug into a toxic derivative (89). MET and ART treatment of C. neoformans each reduced the cytosolic heme pool, presumably by interfering with heme biosynthesis and/or by modifying heme to influence detection by the sensor. These results provide compelling additional support for heme-related activities for the drugs. Importantly, we confirmed that both MET and ART (and the ART derivative DHA) inhibit the proliferation of C. neoformans in culture, suggesting that the drugs are promising leads for repurposed antifungal therapy. Overall, our results support further investigation of heme metabolism as a target for antifungal therapy.

## MATERIALS AND METHODS

### Strains and growth conditions.

C. neoformans var. *grubii* H99 (serotype A) was used as the WT parental strain. Strains were routinely maintained on YPD agar medium (1% yeast extract, 2% Bacto‐peptone, 2% d‐glucose, and 2% agar). Overnight cultures were inoculated with a single colony in liquid YPD medium and incubated at 30°C with shaking at 200 rpm. The vector for the heme sensor (CnHS) (pESL018-2) was biolistically transformed into the WT strain and deletion mutants to generate strains expressing the cytosolic heme sensor. To analyze the heme sensor response to iron conditions, strains expressing CnHS were grown overnight in YPD and iron-starved for 3 h in defined low-iron medium (dLIM; 5 g liter^−1^ glucose, 5 g liter^−1^ l‐asparagine, 0.4 g liter^−1^ K_2_HPO_4_, 0.25 g liter^−1^ CaCl_2_·2H_2_O, 0.08 g liter^−1^ MgSO_4_·7H_2_O, 4.78 g liter^−1^ HEPES, 1.85 g liter^−1^ NaHCO_3_ [dissolved in Chelex 100 resin‐treated water], pH 7.4) containing the iron‐chelator bathophenanthroline disulfonate (BPS; 150 μM). Briefly, the YPD-grown cells (6 × 10^6^ ml^−1^) were inoculated in 100 ml of dLIM with BPS and incubated at 30°C with shaking at 200 rpm for 3 h. After incubation, cells were collected and resuspended in tissue culture-grade phosphate-buffered saline (PBS) at final concentrations of an optical density at 600 nm (OD_600_) 50 ml^−1^ or 1 × 10^9^ cells ml^−1^. The iron-starved cells were used for fluorimetric, flow cytometry, and microscopic analyses as described below. All chemicals were obtained from Sigma‐Aldrich (St. Louis, MO) unless indicated otherwise. The strains, plasmids, and primers used in this study are listed in Table S1 in the supplemental material.

10.1128/mBio.00986-20.8 TABLE S1 Oligonucleotides, plasmids, and strains used in this study. Download Table S1, DOCX file, 0.1 MB. Copyright © 2020 Bairwa et al. 2020 Bairwa et al. This content is distributed under the terms of the Creative Commons Attribution 4.0 International license.

### Construction of the heme sensor for C. neoformans.

The heme sensor probe (CnHS) was designed based on the sequence of the second-generation heme sensor variant HS1-M7A for S. cerevisiae (41). Briefly, a version of HS1-M7A codon optimized for C. neoformans was synthesized (Bio Basic Canada, Inc.) and PCR amplified from plasmid pESL018-1 using the primers CnHS-F and CnHS-R. The resulting fragment (∼1,750 bp) was inserted downstream of the elongation factor 1 promoter (*p*EF1) in a variant of the C. neoformans safe haven vector (pHD58-*p*EF1) previously PCR amplified using the primers pHD58-pEF1-F and pHD58-pEF1-R. The resulting vector pESL018-2 was verified by sequencing. The safe haven vectors are designed to target DNA constructs to the intergenic region of the genes *CNAG_0077* and *CNAG_00778* (90). The vector containing CnHS (pESL018-2) was linearized using AscI for biolistic transformation (Table S1). Transformant strains were selected on hygromycin, and correct genotypes were confirmed by PCR using the primers UQ2962, UQ3348, UQ1768, and UQ2963, as described previously (90).

### Construction of *sod1*Δ and *sod2*Δ deletion mutants.

To construct the *sod1* mutant, a gene-specific knockout cassette was prepared by overlapping PCR using primer pair Sod1KO1-Sod1KO6 (Table S1) with genomic DNA and plasmid pCH233 as the templates. To construct the *sod2* mutant, a gene-specific knockout cassette was prepared by overlapping PCR using primer pair Sod2KO1-Sod2KO6 (Table S1) with genomic DNA and plasmid pJAF1 as the templates. The resulting amplified DNAs were introduced into the WT strain using biolistic transformation. The positive transformants were screened and confirmed by PCR and Southern blot analysis (see Fig. S7).

10.1128/mBio.00986-20.7 FIG S7 Confirmation of *sod1*Δ and *sod2*Δ deletion mutants. Restriction maps and genome hybridization results documenting the replacement of the *SOD1* open reading frame with the NAT resistance marker (A) and the *SOD2* open reading frame with the NEO resistance marker (B). The positions of the probes for hybridization are shown on the maps relative to the positions of the open reading frames and the sites for the restriction enzymes used for genomic digestion (i.e., BspHI and HindIII for *SOD1*, and BglII and XbaI for *SOD2*). The *sod1* mutant 1 and the *sod2* mutant 2 were used in the study. Download FIG S7, PDF file, 0.5 MB. Copyright © 2020 Bairwa et al. 2020 Bairwa et al. This content is distributed under the terms of the Creative Commons Attribution 4.0 International license.

### Microscopy and imaging analysis.

Cells expressing the heme sensor (CnHS) were incubated on different media under conditions at 30°C or 37°C and immediately observed under the fluorescence microscope after the designated incubation time. Differential interference contrast (DIC) and fluorescence imaging were performed with a wide-field fluorescence microscope (Zeiss Axiovert 200) coupled with a CMOS camera (ORCA-Flash4.0 LT; Hamamatsu Photonics) along with a 100× oil immersion lens objective (numerical aperture [NA], 1.40). The eGFP signal was captured with a GFP (excitation [Ex], 470/40; emission [Em], 525/50) filter set at a 0.2-ms exposure time, whereas the mKATE2 fluorescent signal was captured with a mCherry (Ex, 572/25; Em, 629/62) filter set with an exposure time of 1.5 s. Quantification of the mean intensity values of both fluorescent signals was obtained with ZEN Lite 2.3 (version 2.3.69.1000; Carl Zeiss) software, and image analysis was performed with ImageJ and Prism 6 (version 6.01; GraphPad Software).

### Fluorimetric analysis to measure the heme sensor response.

For measurements of the fluorescence response of the heme sensor to varied external hemin concentrations with and without different drugs, WT and deletion mutant strains expressing the sensor were iron starved as described above and collected in tissue culture-grade PBS. Cells (2.5 × 10^6^) were added to a well containing 225 μl of PBS supplemented with different concentrations of hemin (1, 10, 50, and 100 μM) with and without various drug combinations in a 96-well black flat well plate (Greiner Bio-One). The fluorescence signals of eGFP and mKATE2 were recorded on a Tecan Infinite M200 microplate reader using excitation and emission wavelength pairs of 480 nm and 520 nm and 580 nm and 620 nm, respectively, for a period of 2 h at 5-min intervals. Background fluorescence of cells not expressing the heme sensor were recorded and subtracted from the eGFP and mKATE2 fluorescence values.

### Assessing the effects of various inhibitors on heme uptake.

The influences of endocytosis, vacuole physiology, and mitochondrial functions on heme uptake were determined by incubating iron-starved cells (2.5 × 10^6^) expressing the heme sensor in PBS with the inhibitors chlorpromazine (CPZ), monensin, brefeldin A, chloroquine, bafilomycin A, diphenyleneiodonium (DPI), rotenone, salicylhydroxamic acid (SHAM), myxothiazol, antimycin A, or potassium cyanide (KCN) at the designated concentrations with and without hemin (100 μM). eGFP and mKATE2 fluorescence was measured using either fluorimetry or microscopy as mentioned above.

### Flow cytometry analysis.

Flow cytometric measurements of the cells expressing the heme sensor were performed using an Attune NxT acoustic focusing cytometer equipped with an argon laser (Ex, 488 nm) and yellow-green laser (Ex, 561 nm). eGFP was excited using the argon laser and was measured using a 530/30-nm bandpass filter. mKATE2 was excited using the yellow-green laser and was measured using a 610/20-nm bandpass filter. The data were evaluated with FlowJo version 10.6.1 software. The number of cells measured per experiment was set to 30,000, unless otherwise stated. WT cells not expressing the heme sensor were used as a negative control for fluorescence. The analysis was performed only with mKATE2-positive cells.

### Microscopy of macrophage-*Cryptococcus* interactions.

The heme sensor response from WT*^hs^* cells upon phagocytosis by the murine macrophage-like cell line J774A.1 was assessed by performing a macrophage infection assay, as described previously (91). Briefly, J774A.1 cells were cultured at 37°C in 5% CO_2_ in Dulbecco’s modified Eagle’s medium (DMEM) supplemented with 10% heat‐inactivated fetal bovine serum, 1% nonessential amino acids, 100 μg ml^−1^ penicillin-streptomycin, and 4 mM l‐glutamine (Invitrogen). WT*^hs^* cells were grown overnight in YPD medium, opsonized with monoclonal antibody 18B7 against capsule (10 μg ml^−1^; a generous gift from Arturo Casadevall) in serum-free DMEM medium, and added at a multiplicity of infection of 1:10 (macrophage/yeast) to phorbol myristate acetate-activated macrophages. After 2 h of internalization, any extracellular yeast cells were removed by washing with PBS. For microscopic analysis, macrophages were lysed with sterile water to release the phagocytosed yeast cells at the designated times. The yeast cells were collected and resuspended in sterile PBS and observed microscopically to evaluate the eGFP and mKATE2 fluorescence as described above. Microscopic analyses of the cells expressing the heme sensor within the murine macrophages were performed by adding the yeast cells to the activated macrophages grown on a 15-mm glass coverslip in a 24-well tissue culture plate. At the designated times, the coverslip was recovered from the wells and mounted onto a glass slide with DMEM medium without phenol red and immediately observed under a fluorescence microscope.

### Assay of heme content.

To determine total heme content, ∼2 × 10^8^ cells were incubated in 10 ml of PBS supplemented with hemin (100 μM), in the presence or absence of chlorpromazine (CPZ; 100 μM), at 30°C for 1 h and 40 rpm. After CPZ treatment, cells were washed twice with 10 ml sterile water, and the pellets were resuspended in 500 μl of oxalic acid (20 mM) and kept at 4°C in the dark for 16 h. Subsequently, 500 μl of oxalic acid (2 M) was added to the samples, and one-half of the volume of each sample was heated at 95°C for 30 min, while the remaining one-half of the sample was kept in the dark at room temperature for the same duration of time. All the samples were then centrifuged at 16,000 × *g* for 2 min, and 200 μl of the supernatant was collected in a black 96-well plate. The fluorescence measurements (Ex, 410 nm; Em, 610 nm) of the samples were performed in a microplate reader (Tecan Infinite Pro). To determine the intracellular heme content of cells treated with and without MET, ∼2 × 10^8^ cells grown in YPD and YPD-MET (40 and 100 mM) for 16 h were collected and washed twice with sterile water and analyzed using the oxalic acid assay method as described above.

### Drug susceptibility assays.

The influence of MET and ART on growth was examined in liquid YPD medium with different concentrations of drugs either individually or in combination by checkerboard analysis. Briefly, WT cells were grown overnight in YPD, and a total of 2 × 10^6^ cells were added to 180 μl of YPD containing different concentrations of either MET (0, 25, 50, 75, 100, and 200 mM) or ART (0, 0.04, 0.15, 0.6, 2.5, and 10 μg ml^−1^) and up to 30 μg ml^−1^ for DHA alone or in combination in a checkerboard manner to a final volume of 200 μl in a 96-well microtiter plate. The plate was incubated in a Tecan Infinite M200 Pro microplate reader at 30°C and the OD_600_ was recorded for a period of 78 h at 30-min intervals. The growth characteristics of WT cells for the combination of various concentrations of MET and ART (or DHA) were plotted as a heat map from the OD_600_ values recorded at 72 h.

### Serial dilution spot assay.

Cells of the WT or mutants were grown overnight in YPD, inoculated in low-iron yeast nitrogen base (YNB) medium with BPS (150 μM; YNB-Li) and incubated at 30°C with shaking at 200 rpm for 48 h. These iron-starved cells were collected and resuspended in YNB-Li at final concentration of 2 × 10^7^ cells ml^−1^. Tenfold serial dilutions were prepared, and 5 μl of cell suspensions were spotted onto YNB-Li plates containing hemin (10 or 100 μM) with and without CPZ as indicated in the text and figure legends.
