# Supplementary material for: A Cytoplasmic Heme Sensor Illuminates the Impacts of Mitochondrial and Vacuolar Functions and Oxidative Stress on Heme-Iron Homeostasis in Cryptococcus neoformans
Source: mBio. 2020 Jul 28;11(4):e00986-20. doi: 10.1128/mBio.00986-20 (PMC7387795; doi:10.1128/mBio.00986-20)
Supplement: FIG S1 [file mBio.00986-20-sf001.pdf]

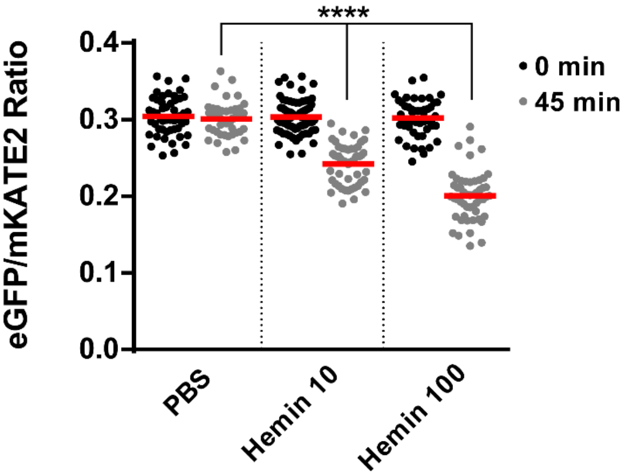

Figure S1. The *CnHS* heme sensor in *C. neoformans* responds to extracellular hemin in a time- and concentration-dependent manner. Heme-dependent change in eGFP/mKATE2 fluorescence ratios of the CnHS in iron-starved WT^{hs} cells incubated with and without hemin (10 and 100 µM) at 30° C for 0 and 45min. Fluorescent values were determined using wide-field fluorescence microscopy observations of the cells (n>50). The data are representative of at least three independent experiments (P-value **** <0.0001, one-way ANOVA followed by Tukey HSD post-hoc test).
