# Supplementary material for: A Cytoplasmic Heme Sensor Illuminates the Impacts of Mitochondrial and Vacuolar Functions and Oxidative Stress on Heme-Iron Homeostasis in Cryptococcus neoformans
Source: mBio. 2020 Jul 28;11(4):e00986-20. doi: 10.1128/mBio.00986-20 (PMC7387795; doi:10.1128/mBio.00986-20)
Supplement: FIG S2 [file mBio.00986-20-sf002.pdf]

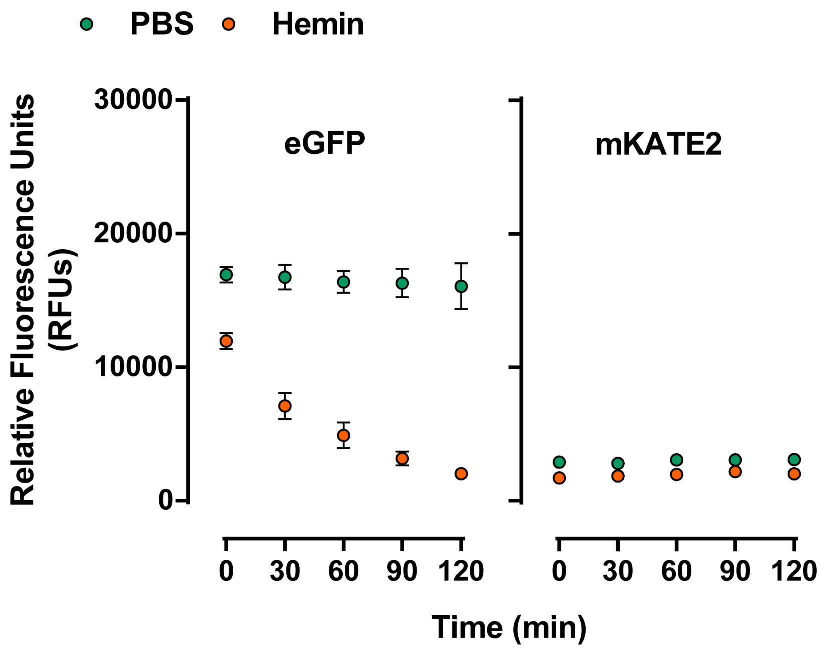

Figure S2. Stability and responses of the fluorescence signals of eGFP and mKATE2 in WT^{hs} cells. The fluorescence signals of eGFP and mKATE2 in WT^{hs} cells incubated in PBS with and without hemin (100 µM) were monitored in a black 96-well plate using a Tecan Infinite® 200 microplate reader for the indicated time points. The data were plotted as relative fluorescence units (RFU) of eGFP and mKATE2 fluorescence after normalization with the background fluorescence of WT cells without the heme sensor. The results are the average of three independent experiments, with the standard error of the mean shown by the bars (not visible for mKATE2 because of the small variation).
