# Supplementary material for: A Cytoplasmic Heme Sensor Illuminates the Impacts of Mitochondrial and Vacuolar Functions and Oxidative Stress on Heme-Iron Homeostasis in Cryptococcus neoformans
Source: mBio. 2020 Jul 28;11(4):e00986-20. doi: 10.1128/mBio.00986-20 (PMC7387795; doi:10.1128/mBio.00986-20)
Supplement: FIG S3 [file mBio.00986-20-sf003.pdf]

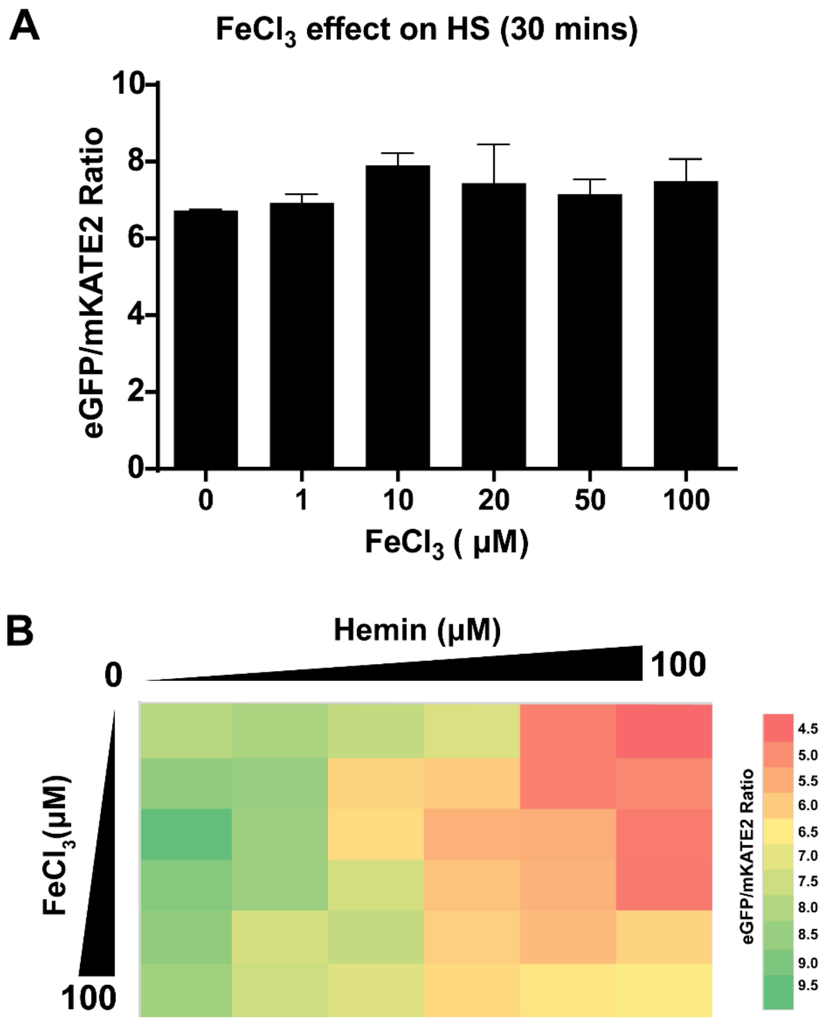

Figure S3. **Inorganic $FeCl_3$ effect on heme sensor response.** (A) eGFP/mKATE2 fluorescence ratios of the CnHS in WT*hs* cells incubated with the indicated concentrations of $FeCl_3$ (0 to 100 μM). The fluorescence for eGFP and mKATE2 was recorded every 5 min and are shown at the 30 min time point. The data were analyzed by averaging the ratios of all the eGFP and mKATE2 fluorescent values, and represent the average of two independent experiments ± SD. (B) eGFP/mKATE2 fluorescence ratios of the CnHS in WT*hs* cells upon incubation for 2 h with different combinations of $FeCl_3$ (0 to 100 μM) and hemin (0 to 100 μM). The heatmap scale depicts the color representation of the eGFP/mKATE2 values for the range of 4.5 to 9.5.
