# Supplementary material for: A Cytoplasmic Heme Sensor Illuminates the Impacts of Mitochondrial and Vacuolar Functions and Oxidative Stress on Heme-Iron Homeostasis in Cryptococcus neoformans
Source: mBio. 2020 Jul 28;11(4):e00986-20. doi: 10.1128/mBio.00986-20 (PMC7387795; doi:10.1128/mBio.00986-20)
Supplement: FIG S4 [file mBio.00986-20-sf004.pdf]

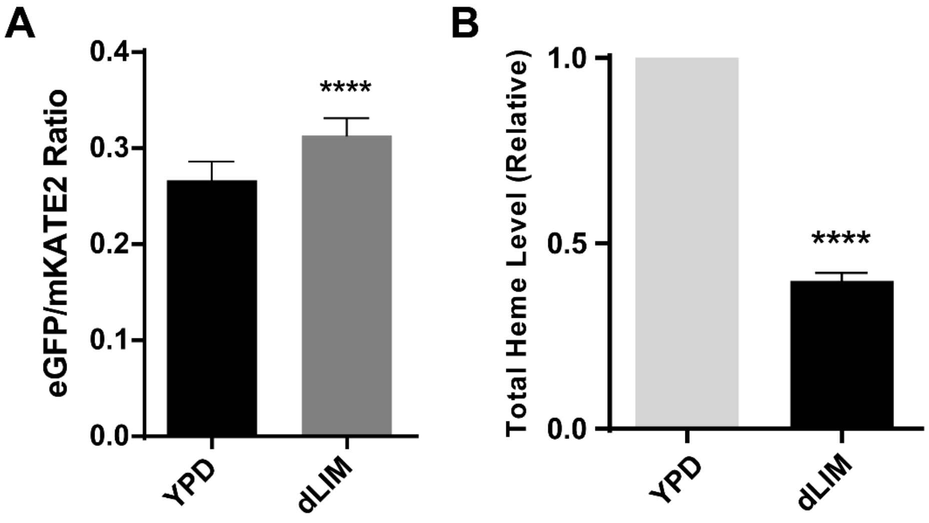

Figure S4. **CnHS response and total intracellular heme levels in rich and low iron media.** (A) Response of CnHS in WT*hs* cells incubated in rich (YPD) and defined low-iron medium (dLIM) with BPS at 30°C for 16 and 3 h, respectively. The data correspond to changes of eGFP/mKATE2 ratios determined using fluorescence microscopy of the cells (n>80) and are representative of three independent experiments with error bars showing ± SD (P-value **** <0.0001, two-tailed Student's *t*–test). (B) Quantification of the total intracellular heme levels in *C. neoformans* wild-type strain grown in YPD and dLIM BPS as in (A). The data represent the average of three independent experiments ± SEM (P-value ** <0.0001, two-tailed Student's *t*–test).
