# Supplementary material for: A Cytoplasmic Heme Sensor Illuminates the Impacts of Mitochondrial and Vacuolar Functions and Oxidative Stress on Heme-Iron Homeostasis in Cryptococcus neoformans
Source: mBio. 2020 Jul 28;11(4):e00986-20. doi: 10.1128/mBio.00986-20 (PMC7387795; doi:10.1128/mBio.00986-20)
Supplement: FIG S5 [file mBio.00986-20-sf005.pdf]

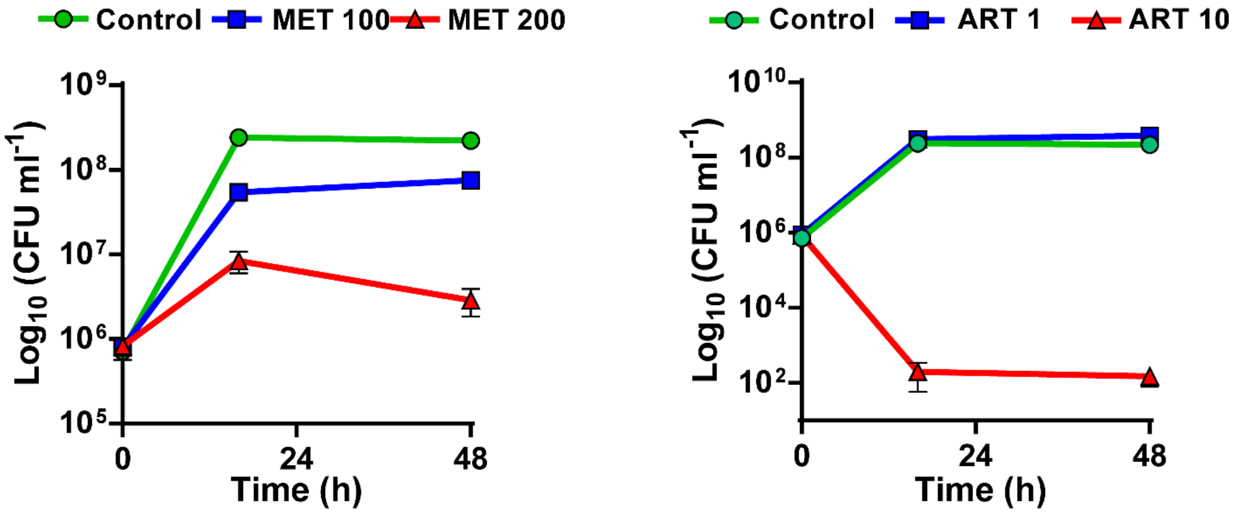

Figure S5. Measurements of fungicidal and fungistatic activity of metformin and artemisinin for *C. neoformans*. WT and WT^{hs} cells were inoculated at 0.1 OD in YPD supplemented with and without metformin (MET, 100 and 200 mM) or artemisinin (ART, 1 and 10 μg ml^{-1}) at 30° C and 200 rpm. Survival was monitored by plating to determine colony forming units (CFUs) at 0, 16 and 48 h. The data represents the average of three independent experiments ± SEM.
