# Supplementary material for: A Cytoplasmic Heme Sensor Illuminates the Impacts of Mitochondrial and Vacuolar Functions and Oxidative Stress on Heme-Iron Homeostasis in Cryptococcus neoformans
Source: mBio. 2020 Jul 28;11(4):e00986-20. doi: 10.1128/mBio.00986-20 (PMC7387795; doi:10.1128/mBio.00986-20)
Supplement: FIG S6 [file mBio.00986-20-sf006.pdf]

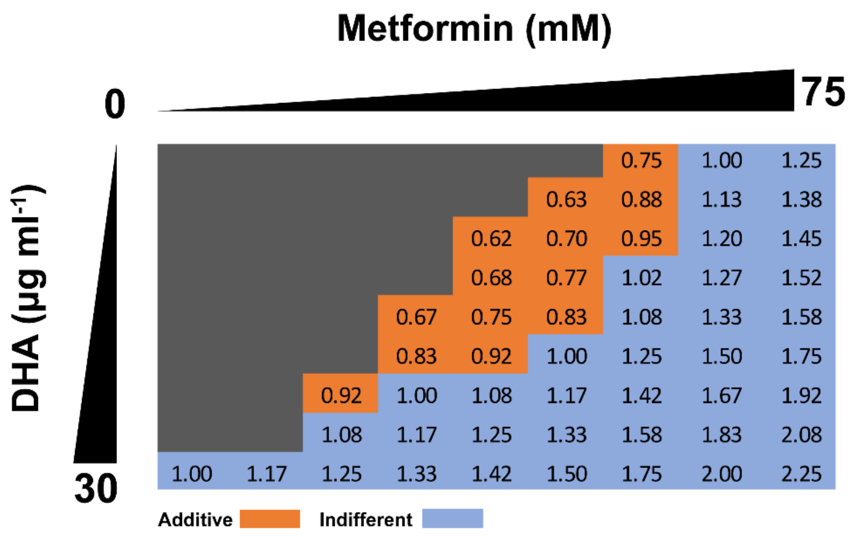

Figure S6. The combination of metformin and the artemisinin derivative DHA exerts an additive or indifferent inhibitory effect on *C. neoformans*. Checkerboard MIC assay of the WT strain showing the Fractional Inhibitory Concentration (FIC) indices of the combinatorial effect of MET (0-75 mM) and the ART derivative dihydroartemisin (DHA, 0-30 μg ml^{-1}). Cells were incubated in a 96 well plate with RPMI medium at ~10^4 cells ml^{-1} for 72 h at 37°C. The MIC values for MET and DHA were 55-60 mM and 28-30 μg ml^{-1}, respectively. FIC indices were defined as: synergism FIC <0.5; additive >0.5 – <1; indifferent ≥1 – ≤2; antagonism >4. The data represent the values of five independent experiments.
