# Supplementary material for: A Cytoplasmic Heme Sensor Illuminates the Impacts of Mitochondrial and Vacuolar Functions and Oxidative Stress on Heme-Iron Homeostasis in Cryptococcus neoformans
Source: mBio. 2020 Jul 28;11(4):e00986-20. doi: 10.1128/mBio.00986-20 (PMC7387795; doi:10.1128/mBio.00986-20)
Supplement: TABLE S1 [file mBio.00986-20-st001.docx]

| **Table S1. Oligonucleotides, plasmids and strains used in this study** | | |
| --- | --- | --- |
| **Oligonucleotides** | | |
| Primer Name | Sequence 5’→3’ | Reference |
| CnHS-F (ES145) | CACATGGTCTCTGAGCTCAT | This study |
| CnHS-R (ES146) | TCAGGACTTGTACAGCTCGT | This study |
| pHD58-pEF1-F (ES148) | GCTGTACAAGTCCTGACTCAGATCCCTAG  ATACATAACTCAG | This study |
| pHD58-pEF1-R (ES147) | GAGCTCAGAGACCATGTGTTTGAAGTTTTC  TGTGGAGATC | This study |
| UQ2962 | GGGTATGCCACAGATGCAGAT | 89 |
| UQ2963 | TTGGATCCTCAATTGTCTCCT | 89 |
| UQ1768 | TCAGCAACGCCGTTGAATCCT | 89 |
| UQ3348 | ACTGGTGAGTACTCAACCAAG | 89 |
| CnHS-seq-1F | CACAGGGGTGTGAGCAAGGG | This study |
| CnHS-seq-2F | GGTGACACCCTCGTCAACCG | This study |
| CnHS-seq-3R | TGGTCTTGAGTTGCTCGGCG | This study |
| CnHS-seq-4R | CCTACCCTCGAGACCACCGT | This study |
| Sod1KO1 | CGCTGGAGGAAGAGAAAGATGATAACGC | This study |
| Sod1KO2 | AATTCTGCAGATATCCATCACACTGGCGG  CTCGGTGGAGTTGTGCCGAGTAAGAAGAC | This study |
| Sod1KO3 | AATTCCAGCACACTGGCGGCCGTTACTAG  TCCATCATTGGTCGAAGCCTCGTCGTCCA | This study |
| Sod1KO4 | CTTGAACCTCCTGAACGGGCTCTG | This study |
| Sod1KO5 | AAACAGGACGAGGCAGAAGATCAG | This study |
| Sod1KO6 | CAATAGGGTCGTTGATAGCGGACATGG | This study |
| Sod1IN_F | GCTGTTGTTGTCCTCAAGGGTGAATC | This study |
| Sod1IN_R | GCCGTAGAGGGAGATGATCTTGT | This study |
| Sod1Southern_F | AGCGGTATTTCTACCTAAGCGCAC | This study |
| Sod1Southern_R | CGTTCCCGCTATTTCTCTTCTCCTC | This study |
| Sod2KO1 | GAGGGATACTCACCCAAGTAGAGAACAGC | This study |
| Sod2KO2 | AATTCTGCAGATATCCATCACACTGGCGGC  GGGAAATAGCAGTCTTGAAGTCGCCATCAG | This study |
| Sod2KO3 | AATTCCAGCACACTGGCGGCCGTTACTAGT  ATTGTCTATCTTCGGGTGTGGCACAG | This study |
| Sod2KO4 | GAGAGTGGAGACGAGAGAAAGCATTG | This study |
| Sod2KO5 | CTCCATAGAGTGTCTCGTCCATAGCCATAC | This study |
| Sod2KO6 | TTACCAGGGCTAACGCCTCGTTACTG | This study |
| Sod2IN-F | CACAAAGGCAGACATATGGTCACC | This study |
| Sod2IN-R | TCCAAATGGCATTGAGGTAGTCGG | This study |
| Sod2_Southern_F | CCAAAATGATCACTGCCATCACTCG | This study |
| Sod2_Southern_R | ATCAGCAGATGCCTTCTGGAGAGAC | This study |
| **Plasmids** | | |
| Name | Description | Reference |
| pESL018-1 | Cloning vector with the *C. neoformans* synthetized codon optimized version of HS1-M7A | This study |
| pHD58-p*EF*1 | HYG vector with elongation factor 1 promoter for targeted integration | unpublished |
| pESL018-2 | Vector with the *C. neoformans* heme sensor (CnHS) for targeted integration | This study |
| pCH233 | Vector harbouring the NAT (nourseothricin acetyltransferase) resistance gene | 91 |
| pJAF1 | Vector harbouring the G418 (Neomycin sulfate analoge) resistance gene | 92 |
| **Strains** | | |
| Name | Description, Genotype | Source, Reference |
| H99/WT | *C. neoformans* wild type strain, serotype A | Joseph Heitman |
| WT*^hs^* | *C. neoformans* wild type strain expressing heme sensor, *pEF1-mKATE2-eGFP-b_562_::HYG* targeted at safe haven. | This study |
| *chc1∆^hs^* | *chc1* deletion mutant in the H99 background with heme sensor, *chc1∆::NAT, pEF1-mKATE2-eGFP-b_562_::HYG* | This study |
| *cig1∆^hs^* | *cig1* deletion mutant in the H99 background with heme sensor, *cig1∆::NAT, pEF1-mKATE2-eGFP-b_562_::HYG* | This study |
| *cfo1∆ cig1∆^hs^* | *cfo1* and *cig1* double deletion mutant in the H99 background with heme sensor, *cfo1∆::NEO, cig1∆::NAT, pEF1-mKATE2-eGFP-b_562_::HYG* | This study |
| *las17∆^hs^* | *las17* deletion mutant in the H99 background with heme sensor, *las17∆::NAT, pEF1-mKATE2-eGFP-b_562_::HYG* | This study |
| *vps45∆^hs^* | *vps45* deletion mutant in the H99 background with heme sensor, *vps45∆::NAT, pEF1-mKATE2-eGFP-b_562_::HYG* | This study |
| *sod1∆^hs^* | *sod1* deletion mutant in the H99 background with heme sensor, *sod1∆::NAT, pEF1-mKATE2-eGFP-b_562_::HYG* | This study |
| *sod2∆^hs^* | *sod2* deletion mutant in the H99 background with heme sensor, *sod2∆::NEO, pEF1-mKATE2-eGFP-b_562_::HYG* | This study |
