# Supplementary material for: A Cytoplasmic Heme Sensor Illuminates the Impacts of Mitochondrial and Vacuolar Functions and Oxidative Stress on Heme-Iron Homeostasis in Cryptococcus neoformans
Source: mBio. 2020 Jul 28;11(4):e00986-20. doi: 10.1128/mBio.00986-20 (PMC7387795; doi:10.1128/mBio.00986-20)
Supplement: FIG S7 [file mBio.00986-20-sf007.pdf]

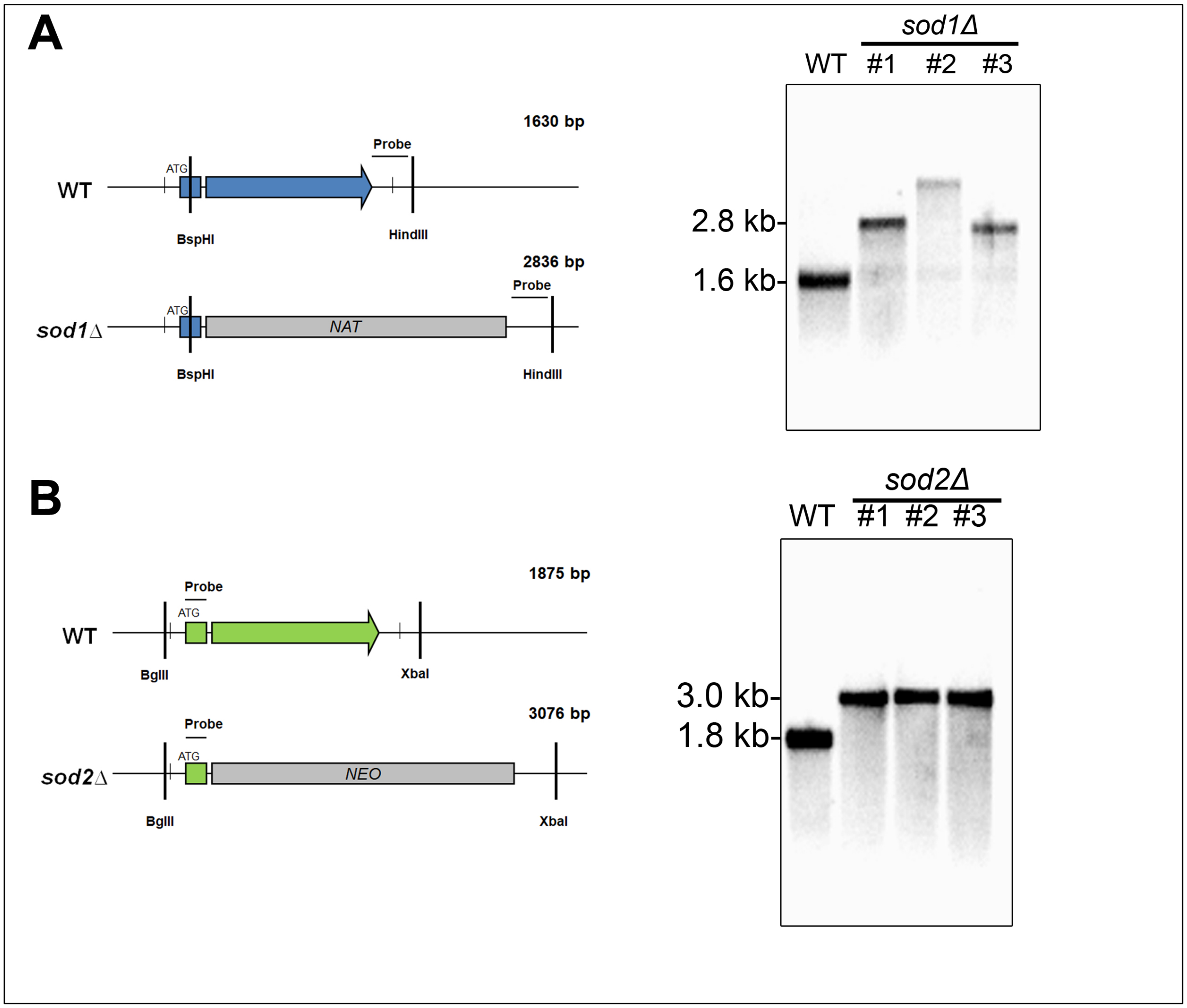

Figure S7. Confirmation of *sod1Δ* and *sod2Δ* deletion mutants. Restriction maps and genome hybridization results documenting the replacement of the *SOD1* open reading frame with the NAT resistance marker (A) and the *SOD2* open reading frame with the NEO resistance marker (B). The positions of the probes for hybridization are shown on the maps relative to the positions of the open reading frames and the sites for the restriction enzymes used for genomic digestion (i.e., BspHI and HindIII for *SOD1*, and BglII and XbaI for *SOD2*). The *sod1* mutant #1 and the *sod2* mutant #2 were used in the study.
